# Infuence of Microstructure in Drug Release Behavior of Silica Nanocapsules

**DOI:** 10.1155/2013/803585

**Published:** 2013-08-06

**Authors:** Gema Gonzalez, Amaya Sagarzazu, Tamara Zoltan

**Affiliations:** ^1^Laboratorio de Materiales, Departamento de Ingeniería de Materiales y Nanotecnología, Instituto Venezolano de Investigaciones Científicas (IVIC), Caracas 20632, Venezuela; ^2^Laboratorio de Fotoquímica, Centro de Química, Instituto Venezolano de Investigaciones Científicas (IVIC), Apartado 20632, Caracas 1020, Venezuela

## Abstract

Meso- and nanoporous structures are adequate matrices for controlled drug delivery systems, due to their large surface areas and to their bioactive and biocompatibility properties. Mesoporous materials of type SBA-15, synthesized under different pH conditions, and zeolite beta were studied in order to compare the different intrinsic morphological characteristics as pore size, pore connectivity, and pore geometry on the drug loading and release process. These materials were characterized by X-ray diffraction, nitrogen adsorption, scanning and transmission electron microscopy, and calorimetric measurements. Ibuprofen (IBU) was chosen as a model drug for the formulation of controlled-release dosage forms; it was impregnated into these two types of materials by a soaking procedure during different periods. Drug loading and release studies were followed by UV-Vis spectrophotometry. All nano- and mesostructured materials showed a similar loading behavior. It was found that the pore size and Al content strongly influenced the release process. These results suggest that the framework structure and architecture affect the drug adsorption and release properties of these materials. Both materials offer a good potential for a controlled delivery system of ibuprofen.

## 1. Introduction

The design of materials for controlled drug delivery has been growing in the last years, due to their importance in the pharmaceutical and health industry. Mesoporous and microporous materials are potentially interesting systems for this purpose due to their high surface area, pore size, structure stability [1, 2], and their characteristics of bioactivity in bone generating implants [3] and biocompatibility [4]. The pore architecture and particle size of the matrix could affect the release profile of the hosted molecules [5–7]. Qu et al. [6] reported that drug loading was directly correlated to surface area, pore geometry, and pore volume in a series of mesoporous materials. Andersson et al. [8] showed that 1D or 3D interconnected pore structures have a strong influence in the release kinetics of the drug. The design strategy for different pore and particle sizes in mesoporous can be approached in different ways, by changing the supramolecular surfactant structure-directing agent or by changing the synthesis conditions. The pH of the synthesis gel strongly affects the hydrolysis-condensation rate of tetraethylorthosilicate (TEOS) and therefore will affect the material geometry modifying the pore architecture, wall thickness and particle size, and the terminal groups located at the walls surface.

Zeolites have a smaller pore size than mesoporous materials, of the order of many pharmaceutical molecules size, and therefore this characteristic could be used to attain a more effective control of the delivery process. In particular zeolite beta is a structure formed by an intergrowth of two or three polymorphs [9, 10] with a pore size of 0.7 nm. The stacking disorder obtained by the presence of different proportions of these polymorphs affects the sorption and desorption properties. 

The pore size of nano- and mesoporous materials to host the guest drug determines the size of the molecule to be adsorbed into the pores. Thus, the adsorption and release of molecules in these matrices are governed by size selectivity, and a pore size of the order of the drug dimension could give a better control of drug release. In the present work, mesoporous materials type SBA-15, synthesized under different pH conditions to obtain different pore sizes, and zeolite beta with different polymorphs proportions were loaded with ibuprofen as drug model, to study how the different morphological aspects (pore size, interconnectivity, and particle size) and Al content affect the drug loading and release processes.

## 2. Experimental Section

The synthesis of the mesoporous materials was carried out using triblock copolymer (EO_20_PO_70_EO_20_) (Pluronic 123), MW = 5800, from BASF, as structure-directing agent. Tetraethylorthosilicate (TEOS), Aldrich, was used as silica source. The EO_20_PO_70_EO_20_ was dissolved in deionized water under continuous agitation for 12 h at room temperature; acid solution of HCl was added until pH cero was reached, and then, TEOS was added under continuous stirring. The variation of pH (0–4.5) was carried out 1 h after the silica hydrolysis began. Hydrothermal synthesis was performed under continuous stirring for 48 h at 90°C. The materials were thoroughly washed and dried at 60°C for 12 h. Calcination was performed at 520°C for 6 h under a constant air flow.

Beta zeolites were synthesized from gels with the following molar composition: Al_2_O_3_ : *x*SiO_2_ : *y*TEA_2_O : 15*x* : H_2_O, with SiO_2_/Al_2_O_3_ ratio = 100 and different TEA_2_O/SiO_2_ ratios of 0.27, 0.50, and 0.75. Crystallization was carried out without agitation at 140°C in a stainless steel reactor. The solids obtained were separated by centrifugation, washed with distilled water, and calcined at 600°C for 12 h, under constant air flow. 

The drug was loaded by the immersion of the calcined mesoporous and beta zeolites samples in ibuprofen (IBU) hexane solution 10^−3 ^M. The procedure of loading ibuprofen was by impregnation, using 100 mg of the synthesized materials in 25 mL of ibuprofen hexane solution, under continuous stirring for 1 to 24 hours. The amount adsorbed and released was monitored by UV-vis spectroscopy for different periods. The drug-loaded sample was separated from the solution by filtration and washed with hexane. This solid was analyzed by TGA in a temperature range of 30 to 900°C. The drug release was followed by placing the loaded materials into a simulated body fluid, SBF [11], at 37°C and at physiological pH of 7.4, maintaining the ratio SBF volume (mL) per adsorbed ibuprofen mass (mg) equal to 1. Continuous magnetic stirring was maintained during the delivery experiments, to avoid limitation of the delivery rate by external diffusion constrains. The loaded ibuprofen concentration was monitored by UV-vis spectroscopy at a wavelength of 272 nm and the delivered ibuprofen at 222 nm. All samples were measured by triplicate and average values were used for the graphical presentation and data treatment. The standard deviations are less than 5% in all cases. 

Characterization of meso- and microporous materials was carried out by X-ray diffraction (XRD), in a Siemens 5005 X-ray diffractometer, using Cu-K*α* (Ni filter) operating at 40 keV and 20 mA. Fourier Transformed Infrared spectroscopy (FTIR) was performed in a Nicolet 560 equipment, scanning electron microscopy (SEM) analysis in a Hitachi FE S-4500 operating at 8 and 10 keV, transmission electron microscopy (TEM) in a Phillips CM-10 operating at 80 keV, and a Tecnai G20 FEI, superficial area measurements by N_2_ adsorption were taken in a Micromeritics ASAP 2010, previously degassing of the samples was performed at 320°C, for 4 h for the unloaded samples, and at 100°C, for 24 h for the drug-loaded samples. Thermogravimetric analyses were carried out in an SDT Q600 TA Instruments equipment, using a heating rate of 10°C/min, from 30 to 900°C, in air atmosphere. The UV experiments were carried out in a Perkin Elmer Lamda2 UV spectrometer. 

## 3. Results and Discussion

**Figure 1:**
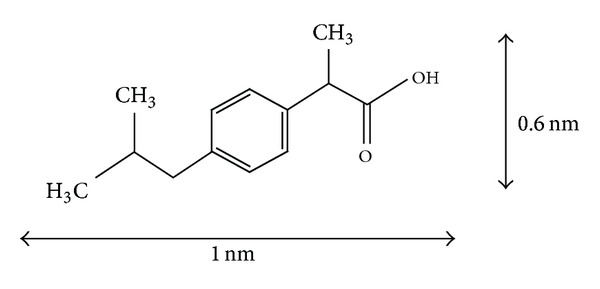
Ibuprofen molecule.

Ibuprofen is used as analgesic and anti-inflammatory and in general acts as a vasoconstrictor; its molecular size is 1.3 × 0.6 nm (Figure 1). It is used as a model molecule in experiments of controlled drug release, due to its stability, its applicability, and its well-known behavior. Due to its dynamical diameter sizes, this molecule is interesting to compare the drug adsorption and release capability of solids of very different pore size, such as zeolites (with a pore size of 0.7 nm) and mesoporous materials with pore size of 50 nm. This can give information of the accessibility of this molecule to the pore channels and therefore evaluate the potential of this solids to be used as drug carriers.

Therefore, mesoporous SBA-15 materials, with different pore size and beta zeolite, were used as nanocapsules. They are both silica based materials with silanol groups in their internal and external surfaces, that can interact with the carboxylic acid groups of IBU via hydrogen bonding or with the pi electron density of the aromatic ring.

**Figure 2:**
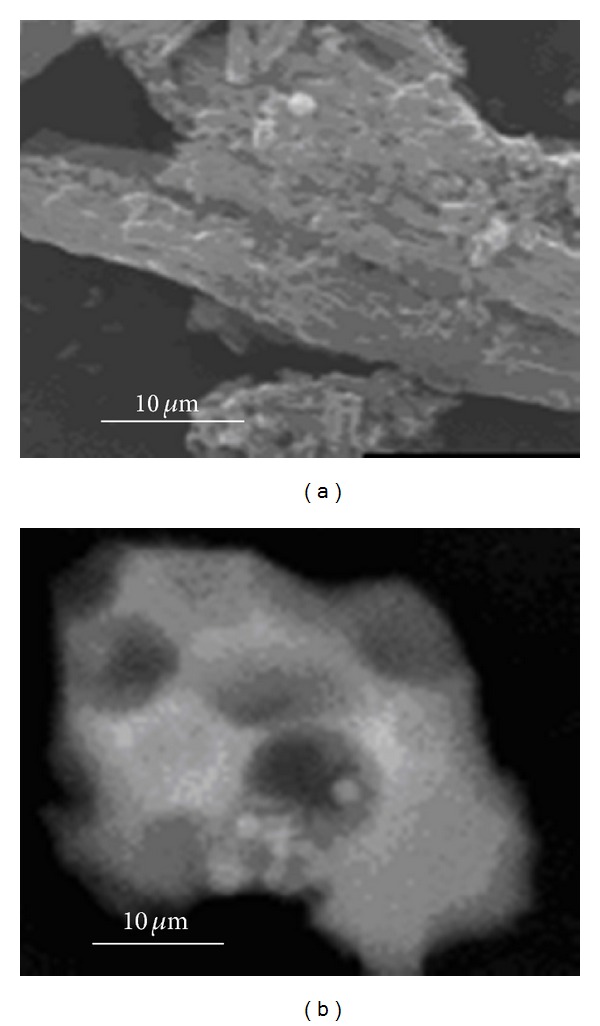
SEM images of SBA-15 synthesized at different pH: (a) pH 0 (SBA_pH  0_); (b) pH 4.5 (SBA_pH  4.5_).

The pore architecture of the mesoporous materials was modified by control of the pH of the synthesis gel, working in conditions above and below the isoelectric silica point.  Figure 2 shows the dramatic change of particle morphology by SEM, for the materials synthesized at pH 0 and 4.5, identified as SBA_pH  0_ and SBA_pH  4.5_. The formation of macropores and the reduction of particle size as pH increases can be observed. This can be attributed to several parameters that influence the hydrolysis and condensation reactions: activity of metal alkoxide, water/alkoxide ratio, solution pH, temperature, and nature of the solvent and additive [12]. 

**Figure 3:**
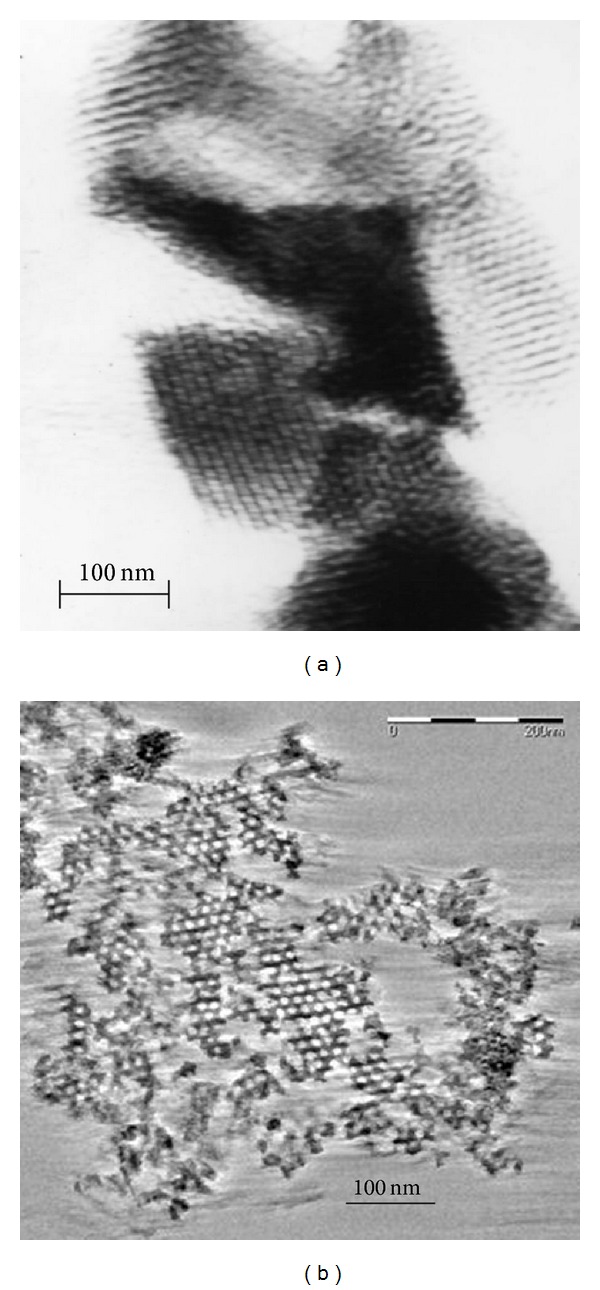
TEM images of SBA-15 synthesized at different pH: (a) pH 0 (SBA_pH  0_); (b) pH 4.5 (SBA_pH  4.5_).

The microstructure and surface chemistry of these materials are very sensitive to variation of these parameters. Also, preservation of the long range hexagonal order of the mesopores is maintained with the change in pH, as shown in Figure 3. 

**Figure 4:**
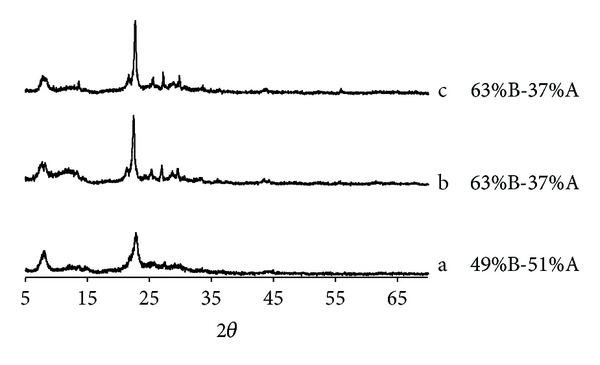
XRD patterns of different beta zeolites synthesized with different TEA_2_O/SiO_2_ ratios: (a) 0.27, (b) 0.75, and (c) 0.5, with proportion of polymorphs indicated on the right hand side.

Beta zeolites with different polymorphs proportions and different Al content were also studied. The XRD of the three zeolites synthesized with different conditions is shown in Figure 4, indicating that the pure phase was obtained. These XRD patterns show, simultaneously, broad and sharp peaks, indicating the highly disordered structure present, typical of these kinds of materials [13]. The characteristic peaks at 2*θ* = 7.8° and 22.5°, assigned to (001) and (302) planes, respectively [14], are observed. The broad peak in 2*θ* = 7.8° is related to the defect structure, while the sharp peak in 2*θ* = 22.5° is attributed to the crystallinity of the samples. The different synthesis conditions using TEA_2_O/SiO_2_ ratios 0.27, 0.5, and 0.75 resulted in solids with different polymorphs proportions and different Si/Al ratios. The determination of the relative amount of polymorphs present was carried out using the program DIFFaX [15]. The different proportions of polymorphs in the structure and Al content could result in different diffusion paths that can affect the adsorption and releasing of the drug.

**Figure 5:**
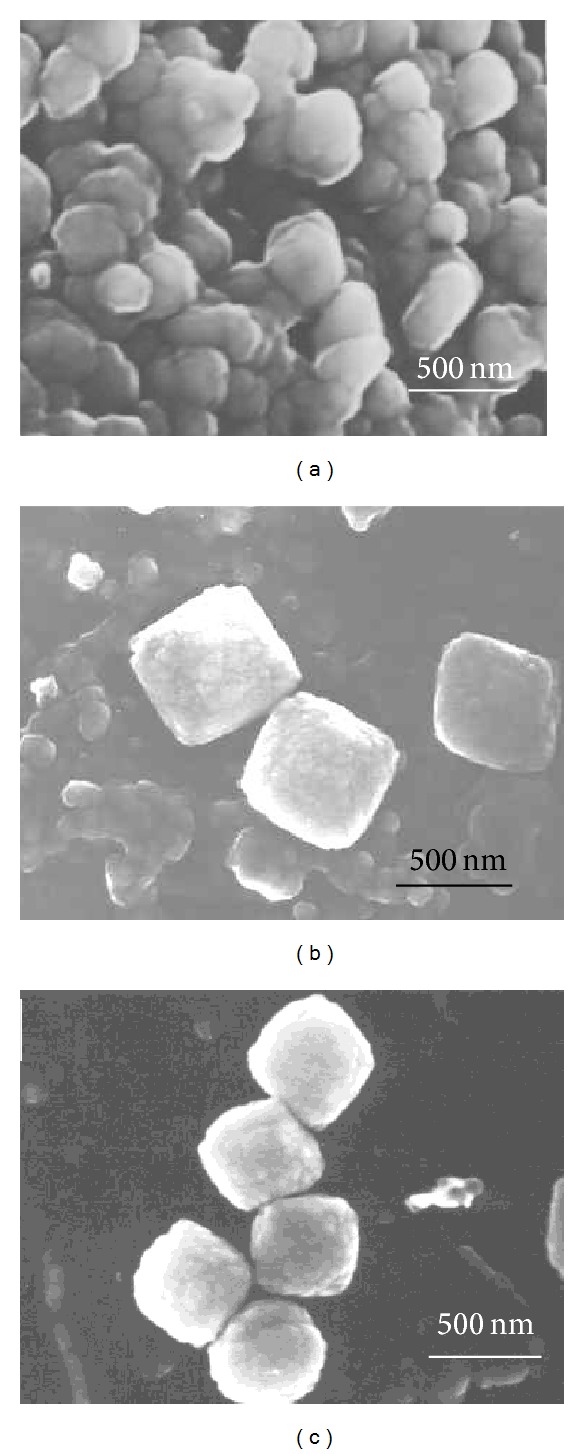
SEM images of zeolites with Si/Al: (a) 28, (b) 10, and (c) 100, respectively.

The different synthesis conditions resulted in zeolites with different morphologies and particle sizes, Figure 5 shows SEM images of the three beta zeolites obtained.  Figure 5(a) (sample a) shows crystals of approximately 200 nm with oval morphology, corresponding to a polymorph proportion of 49% B-51% A and Si/Al = 28, and Figure 5(b) (sample b) and Figure 5(c) (sample c) present crystals of around 400 nm and cuboid morphology with polymorph proportion of 63% B-37% A and Si/Al ratios equal to 10 and 100, respectively. Therefore, zeolites with different crystal sizes and different polymorph proportions and zeolites with the same crystal size and similar polymorph proportion but different Al content are compared.

**Figure 6:**
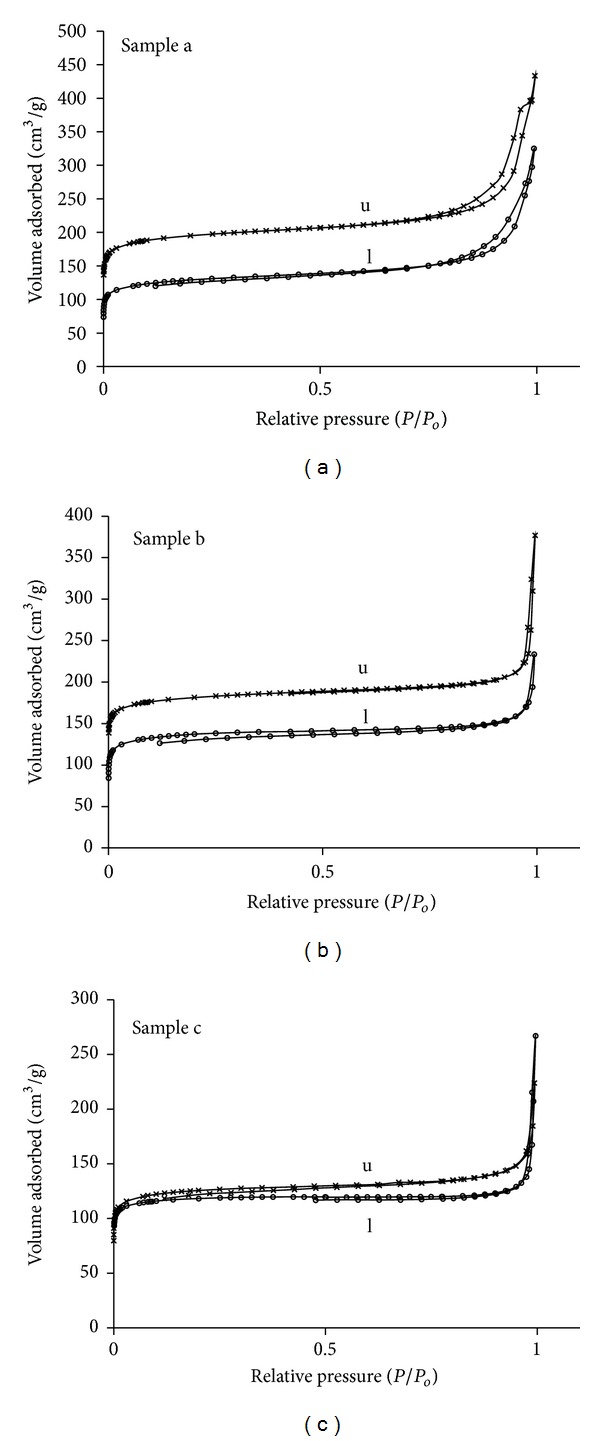
N_2_ adsorption isotherms of beta zeolites with different Si/Al ratios, unloaded (u) and IBU loaded (l).

The N_2_ isotherms of the different zeolite materials calcined and loaded with ibuprofen are shown in Figure 6; the superficial area and pore volume are given in Table 1. These samples showed a typical type I isotherm with surface area and micropore volume characteristic of these materials. It can be observed that the region of low relative pressure, corresponding to zeolites microporosity, decreases with IBU loading indicating a reduction on superficial area of approx. 30% for all the samples, suggesting that either part of the pores is occupied by the drug molecule or IBU molecules are occluding the pore entrance. Also, the decrease in pore volume varied for each sample, implying a different drug adsorption mechanism for each zeolite. It can be observed that the sample with higher Al content (sample b) presents a lower decrease in pore volume than the samples with lower Al content (samples c  and a), as expected. The adsorption capacity of zeolites should increase with the increase in their hydrophobic character, and this is dependent on the Al content, increasing as the Al content decreases. Therefore, it would be expected that the loading of a hydrophobic drug should be higher in sample c, being the sample with the highest hydrophobic character. Sample b showed the lowest reduction in pore volume attributed to pore entrance blocking due to the presence of extraframe Al, implying that the drug does not completely fill all the micropore system but it is also adsorbed on the external surface. In addition, it must be considered that the molecular size of the van der Waals surface of ibuprofen is 1.3 × 0.6 nm^2^ and the reported pore size of zeolites beta is 0.7 nm; therefore some molecules might experience a steric hindrance to enter into the pore space available and probably most of them are located in the outer surface of these materials.

On the other hand, the structural differences between these samples could also have an effect on drug adsorption; sample a has smaller crystal size and higher content of polymorph A (49% B-51% A) compared to samples b and c (63% B-37% A). Polymorphs A and B have similar structures, even though polymorph A is somewhat more tortuous than polymorph B, and the net tortuosity could affect accessibility to the pore system. This could be one of the reasons that sample a shows a lower pore volume decrease, after drug loading, than sample c (Table 1), besides its less hydrophobic character due to the higher Al content.

**Figure 7:**
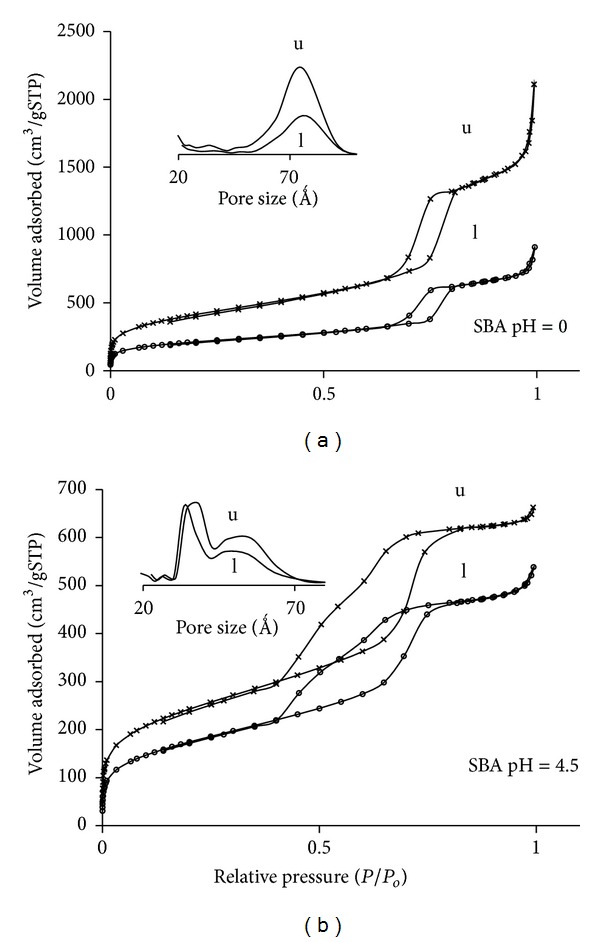
N_2_ adsorption isotherms of SBA-15 mesoporous materials synthesized at different pH, unloaded (u) and IBU loaded (l).

The N_2_ adsorption isotherms and BJH pore size distributions of the mesoporous materials synthesized at the different pH are shown in Figure 7 and Table 2. The isotherms are type IV and exhibit hysteresis loops with well-defined adsorption and desorption branches. Some differences in the shape of the hysteresis loop, especially in the desorption branches, for each pH condition are observed. This variation is attributed mainly to changes in the pore morphology and pore size distribution. A decrease in pore size is observed with increase in pH.

The material synthesized at pH = 0 has a BET surface area of 1288 m^2^/g, a mesopore volume of 2.92 cm^3^/g, and a micropore volume of 0.12 cm^3^/g showing a narrow pore size distribution with a mean value of 78 Ǻ, according to BJH model. This material showed a hysteresis loop H1 type [16] indicative of open cylindrical mesopores with a narrow pore size distribution. This is consistent with the typical well-ordered 1D cylindrical channels forming a hexagonal arrangement characteristic of SBA-15. The material synthesized at pH 4.5 showed a BET surface area of 742 m^2^/g, a mesopore volume of 0.80 cm^3^/g, a micropore volume of 0.09 cm^3^/g, and a bimodal pore size distribution centred at 38 and 55 Ǻ. The hysteresis loop of this sample presents a stepwise desorption isotherm, suggesting the presence of energetically different sites, consistent with the bimodal pore size distribution. As pH increases, the shape of the loop changes [16], suggesting a more random distribution of pores and probably an interconnected pore system. At pH = 0 the silica gel is below the isoelectric point and therefore hydrolysis dominates, while at pH = 4.5 it is above the silica isoelectric point where condensation dominates [17], resulting in a wider pore size distribution. These results indicate that the pH of the synthesis gel strongly affects the mesostructure. The particle size of these materials is also very different (around 50 *μ*m for SBA_pH  0_ and 20 *μ*m for SBA_pH  4.5_).

The IBU loaded mesoporous materials showed a large reduction in surface area and meso- and micropore volumes with respect to the unloaded materials (Table 2). This effect is more pronounced in the material synthesized at pH = 0, that showed a decrease in surface area of 48% and a mesopore volume reduction of about 50%, while the material synthesized at pH = 4.5 only showed a surface area reduction of 26% and a mesopore volume reduction of 20%. Therefore, only a portion of the channels are filled with the drug. The ibuprofen molecules do not fully occupied the available space.

**Figure 8:**
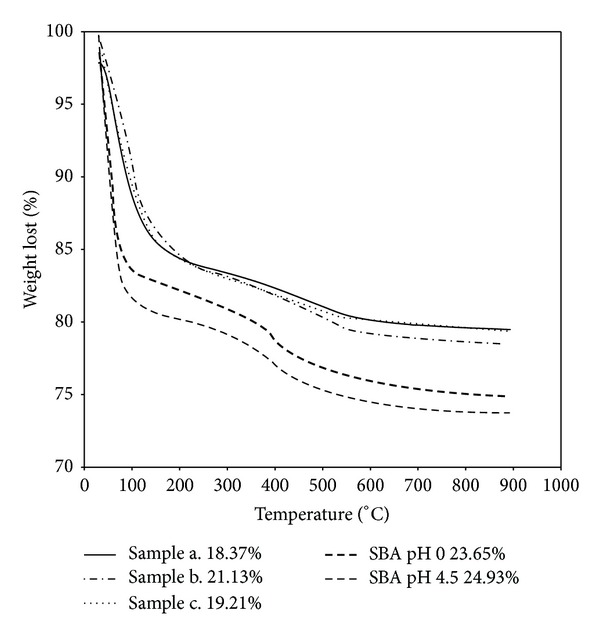
TGA IBU loading of the different materials studied.

**Figure 9:**
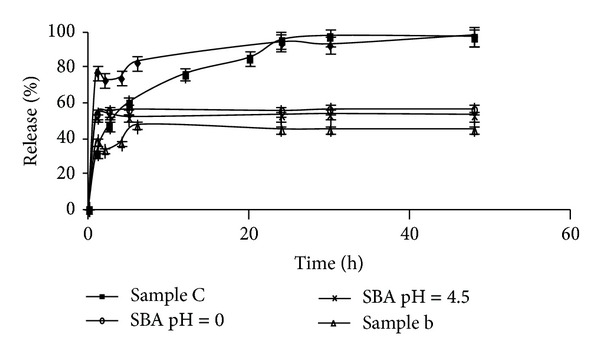
Cumulative release rates of ibuprofen in simulated body fluid.

Thermal gravimetric analysis (TGA) has been used to determine the degree of loading of ibuprofen for the different materials studied; a good correspondence was obtained between this technique and UV-Vis spectrophotometry (Table 3 and Figure 8). In general, the IBU adsorption was very similar for all micro and mesoporous materials (Table 3). However, the delivery rate of IBU is different for each material (Figure 9); probably this is related to the specific structural and surface characteristics of each material. Specially, among the zeolites studied the delivery behavior was different. The amount of ibuprofen released after 7 h was 80% for sample a, 60% for sample c, and 45% for sample b. This implies that the diffusion process in sample a is very fast, hence controlled by external diffusion; while sample c presents a slow delivery rate up to 24 h, when the maximum drug load has been released; therefore a mixture of external diffusion and diffusion through the pores can be present. In samples b and a slow delivery is observed up to 7 h with a 45 wt% of the loaded drug released, and then a stationary stage was reached. This behavior is probably due to the presence of extraframe Al in this material, forming a strong interaction with the carboxylic groups of ibuprofen. It has been reported that carboxylic acids adsorbed in aluminum oxide surfaces [18–20] and in dealuminated FAU [7] are in the form of carboxylate species and the drug was present as ibuprofenate coordinately bonded to extraframework Al species. Therefore, the adsorption of the drug on the surface is stronger for materials with high Al content, leading to a slower delivery in the media, as it has been observed for zeolite sample b (higher Al content). For sample c (lower Al content) due to its hydrophobic character, the drug molecule probably diffuses into the zeolite channels and van der Waals interactions become important to retain the ibuprofen molecules; this could explain the slower drug delivery rate observed in this sample during the first 24 h (Figure 9).

In the mesoporous materials the drug adsorption of both materials was slightly different. The SBA_pH  0_ showed a loading degree of 21.33%, and 25.77% for SBA_pH  4.5_; these values were determined by UV-Vis spectrophotometry, in good agreement with the values reported in the literature for these materials [8, 18] and very similar to the amount adsorbed by the zeolite materials (Table 3). In order to understand the differences in drug adsorption between both mesoporous materials, the amount of ibuprofen adsorbed per gram of material was calculated (Table 3). The values obtained at maximum loading were 10.7 mg/g for SBA_pH  0_ and 12.9 mg/g for SBA_pH  4.5_. The larger reduction in superficial area and pore volume observed, after drug loading, can be attributed to IBU adsorption mainly on the micropores of these materials.

The IBU release *in vitro* process (in SBF) is presented in Figure 9 and Table 3, showing a very similar delivery pattern for SBA materials. They show a fast drug release in the initial periods, and after only 1 h a stationary stage is reached, but only releasing 58% of the loaded drug, even after long periods. The ibuprofen molecular size (1.3 × 0.6 nm^2^) is small compared to the mesopores size of both SBA materials. The free spaces available, in these open cylindrical pores, do not present any diffusion impediment, favoring drug transport from the pores to the solution. However, the drug is not completely released probably due to H bonding between the carbonyl moiety of the carboxylic acid and the silanol groups present on the silica surface. This could be responsible for the anchoring of the IBU molecules. Probably prolonged drug delivery periods will result in complete release, as has been reported by Andersson et al. [8] studying drug release profiles in MCM materials. 

The controlled release of ibuprofen has also been studied through the interpenetrating network of different polymeric microgels of sodium alginate and acrylic acid [19, 20]. For these materials it was reported that a 70% drug release was reached after 6 h and between 85 and 100% after 12. In our case for the zeolitic materials a 100% drug release was achieved after 30 h and for the SBA materials complete drug release was not accomplished even after 100 h, suggesting that for long treatments these materials could probably be more effective in bone tissue applications due to their bioactive character.

## 4. Conclusions 

The amount of ibuprofen loaded in all the different micro- and mesoporous materials is very similar, and it was independent of crystal size, pore size, pore volume, superficial area, and Al content. The release process was affected by these parameters, and zeolites with low Al content showed slow release process in the first hours and then the load was completely released after 24 h. However zeolites with high Al content did not completely release the full amount of loaded drug only 60% was delivered after 72 h this was attributed to the strong interaction ibuprofen with Al through ibuprofenate species. In the mesoporous materials, drug delivery was fast in the first hour and then a steady state was reached and the total drug release was only 58% of the adsorb drug. This is probably due to van der Waals interaction between the carboxylic groups and the silanol surface groups. Both materials have the capability of acting as convenient reservoir for controlled IBU delivery.

## Figures and Tables

**Table 1:** Textural properties of beta zeolites unloaded and IBU.

Material	Loading condition	BET area m^2^/g	Δ Area (%)	Langm. area (m^2^/g)	Δ area (%)	Micro. vol. (cm^3^/g)	Δ pore vol (%)	Micropore area (m^2^/g)	Ext. area (m^2^/g)	Part. size (nm)	Si/Al
Zeolite beta sample a	Unloaded	660	27	946	36	0.27	37	584	77	200	28
	IBU loaded	479		602		0.17		415	64		

Zeolite beta sample b	Unloaded	513	27	707	14	0.23	9	494	28	500	10
	IBU loaded	373		616		0.21		349	19		

Zeolite beta sample c	Unloaded	443	30	588	30	0.19	47	392	51	500	100
	IBU loaded	310		411		0.10		275	23		

**Table 2:** Textural properties of SBA materials synthesized at different pH, unloaded and IBU loaded.

Material	Loading condition	BET area (m^2^/g)	Micropore volume (cm^3^/g)	Mesop. volume (BJH-cm^3^/g)	Pore diameter (Å)	Particle size (*μ*m)
SBA-15 (pH = 0)	Unloaded	1288	0.12	2.92	78	80
	IBU loaded	678	0.10	1.35	78	

SBA-15 (pH = 4.5)	Unloaded	742	0.09	0.80	38–55	25
	IBU loaded	549	0.03	0.68	33–54	

**Table 3:** Loading degree of ibuprofen determined by UV and TGA for the different micro- and mesoporous materials.

Material	Loading degree (wt%) UV	Loading degree (wt%) TGA	mg IBU/g sample	IBU ads/Vpore mmol IBU/cm^3^
Zeolite beta—sample a	20.82	18.37	10.40	0.19
Zeolite beta—sample b	23.15	21.13	11.58	0.24
Zeolite beta—sample c	21.93	19.21	10.98	0.28
SBA 15 (pH = 0)	21.33	23.65	10.67	0.02
SBA 15 (pH = 4.5)	25.77	27.98	12.89	0.08
